# Postresuscitation platelet transfusion in major trauma patients

**DOI:** 10.1111/trf.18414

**Published:** 2025-09-19

**Authors:** Andrea Rossetto, Joseph Reynolds, Ella Ykema, Ross Davenport, Elaine Cole, Paul Vulliamy

**Affiliations:** ^1^ Centre for Trauma Sciences, Blizard Institute Queen Mary University of London London UK; ^2^ Barts Health National Health Service Trust London UK

**Keywords:** critical care, mortality, platelet transfusion, thrombocytopenia, trauma

## Abstract

**Background:**

Platelet transfusions (PLT‐t) are a cornerstone of contemporary trauma resuscitation, but little is known about their use in the postresuscitation period. Our aims were to describe the utilization of PLT‐t after resuscitation and examine their impact on platelet count and clinical outcomes.

**Study Design and Methods:**

Adult trauma patients admitted to critical care at a single major trauma center were included. We compared patients who received PLT‐t postresuscitation (>24 h after injury) with those who did not and examined platelet increments before and after each individual PLT‐t episode. Logistic regressions were constructed to examine the association between postresuscitation PLT‐t and clinical outcomes.

**Results:**

This study included 803 injured patients, of whom 109 (14%) received at least one PLT‐t after resuscitation. Overall, 30% (221/725) of all platelet units administered to the cohort were given in the postresuscitation phase, most in the first week of admission and to patients with moderate–severe thrombocytopenia. The median platelet count increment following transfusion was 19 × 10^9^/L (interquartile range 1–30), and 24% of transfusions failed to increase the platelet count within 24 h. Postresuscitation PLT‐t in patients with moderate–severe thrombocytopenia was independently associated with reduced mortality (OR 0.42, *p* = .039) but a longer critical care length of stay among survivors (coefficient 0.35, *p* = .007).

**Conclusion:**

Postresuscitation PLT‐t is frequently administered in trauma patients. The impact on platelet counts is variable, likely attributable to differences in timing and pretransfusion platelet count. After adjusting for relevant confounders, postresuscitation PLT‐t was associated with reduced mortality in this cohort.

**Abbreviations**
ACIT‐II: Activation of Coagulation and Inflammation in Trauma
Adj.: adjusted
AIS: abbreviated injury severity score
CI: confidence interval
Coef.: coefficient
CTCOFR: complete organ failure recovery
EXTEM: tissue factor–activated rotational thromboelastometry
FIBTEM: tissue factor–activated rotational thromboelastometry with cytochalasin D
IQR: interquartile range
ISS: injury severity score
MODS: multiorgan dysfunction syndrome
mSOFA: modified sequential organ failure assessment
OR: odds ratios
PLT‐t: platelet transfusions
RBC: red blood cells
ROTEM: rotational thromboelastometry
RRT: renal replacement therapy
SOFA: sequential organ failure assessment

## 1 INTRODUCTION

Platelet transfusions (PLT‐t) are a central component of modern hemostatic resuscitation in bleeding trauma patients. Evidence for their use in this setting comes from a range of large‐scale observational studies^1^ as well as randomized trials,^2^, ^3^, ^4^ where higher volumes of PLT‐t given early after injury have been linked to reduced mortality. As such, current guidelines support their administration as part of major hemorrhage protocols in a 1:1:1 ratio with red blood cells (RBCs) and plasma.^5^, ^6^ However, there is a lack of published data on PLT‐t given to trauma patients after the resuscitation phase is complete. Specifically, little is known about the relationship between postresuscitation PLT‐t and clinical outcomes, their impact on platelet count trajectories, and the resource implications associated with their use.

In nontrauma settings, there is considerable controversy and uncertainty around the indications for and efficacy of PLT‐t. Randomized trials of neonatal thrombocytopenia and antiplatelet‐associated intracerebral hemorrhage^7^, ^8^ found that PLT‐t resulted in worse clinical outcomes, findings that are echoed by observational data in disorders of platelet consumption.^9^ The mechanisms underlying these observations are not clear but may relate to the diverse roles of platelets beyond hemostasis, such as their immunomodulatory effects.^10^ The impact of PLT‐t given after the initial hemorrhage phase on clinical outcomes after injury has not been specifically examined.

In the critical care setting, the recent PLOT‐ICU study and a previous cross‐sectional study highlighted significant variability in PLT‐t practice, with less than a quarter of patients with thrombocytopenia receiving a PLT‐t and with significant variation in pretransfusion platelet count.^11^, ^12^ However, these studies included small numbers of injured patients and did not examine the need for or impact of PLT‐t according to the underlying pathology. We have recently shown that trauma patients have a high incidence of thrombocytopenia during postinjury critical care admission,^13^ and trauma patients frequently require multiple surgical interventions during their hospital stay that require optimization of hemostasis. We hypothesized that postinjury PLT‐t would be a frequent occurrence and a major contributor to overall transfusion‐related resource requirement in this patient group.

The aim of this study was to describe the utilization of PLT‐t in the postresuscitation phase after severe injury and investigate associations with clinical outcome. Our specific objectives were to quantify the need for PLT‐t after resuscitation, determine the impact on platelet count trajectories, and characterize the association between postresuscitation PLT‐t and mortality.

## 2 METHODS

### 2.1 Study design

In this study, we included adult patients (≥16 years) who required the activation of the trauma team and were enrolled in the perpetual prospective observational cohort study Activation of Coagulation and Inflammation in Trauma (ACIT‐II, ISRCTN12962642). This ACIT‐II study was approved by the East London and The City Research Ethics Committee (07/Q0603/29). All procedures involving human subjects were performed in accordance with the 1964 Helsinki Declaration and its later amendments or comparable ethics. Patients were excluded if the time from injury to admission was >2 h, more than 2000 mL of fluid was administered prehospital, patients suffered burns >5% body surface area, or refused consent. Consent procedures for the ACIT‐II study have been previously reported in detail.^14^ For the purpose of this study, we specifically included all patients admitted to critical care (high dependency unit or intensive care unit) from January 2014 to December 2023, and who had at least one measurement of platelet count during their critical care stay.

### 2.2 Data collection

Trained member of the research team prospectively collected data from patients' admission (day 0) until day 28 after injury, or until discharge or death if these occurred earlier. The variables collected included demographics, injury characteristics, admission vital signs and laboratory parameters, platelet count, sequential organ failure assessment (SOFA) component scores during critical care stay, need for invasive ventilation, vasopressor support, and renal replacement therapy (RRT), incidence and type of venous thromboembolic events, critical care length of stay, and mortality.

### 2.3 Blood sampling and analysis

Blood samples for research purposes were drawn together with clinical blood samples upon admission. The latter were also collected during the critical care stay according to the clinicians' directives. Platelet count was measured in the hospital laboratory and base deficit by point‐of‐care blood gas analysis according to standard operating procedures. Rotational thromboelastometry (ROTEM) was performed using ROTEM Delta analyzers (TEM Innovations GmbH) within 1 h after blood sampling. Tissue factor‐activated ROTEM (EXTEM) and tissue factor‐activated ROTEM with cytochalasin D (FIBTEM) were stopped automatically after 60 min. Clot amplitude at 5 min (A5) was used in this study. Platelet contribution was calculated as the difference between EXTEM and FIBTEM values (EXTEM–FIBTEM).^15^ ROTEM methodology and parameters have been previously described in detail.^16^

### 2.4 Study outcomes

Our primary outcome was in‐hospital mortality. Secondary outcomes were critical care length of stay and prolonged organ support as measured by the composite time to complete organ failure recovery (CTCOFR) in survivors.^17^, ^18^ We also reported venous thromboembolism and multiorgan dysfunction syndrome (MODS), as measured by the SOFA score. As previously described, to exclude the confounding impact of the hematological component (platelet count), we modified the SOFA score (mSOFA) by removing this variable in some analysis.^19^, ^20^

### 2.5 Definitions

Thrombocytopenia was defined as a platelet count of <150 × 10^9^/L and classified as mild (100–149 × 10^9^/L), moderate (50–99 × 10^9^/L) and severe (<50 × 10^9^/L) as previously reported.^11^ PLT‐t episodes were defined as the intervals between platelet count measurements during which PLT‐t were administered. The injury severity score (ISS) and admission base deficit were used as markers of injury burden and shock, respectively. Traumatic brain injury was defined as a head abbreviated injury score ≥3. Coagulopathy was defined as EXTEM A5 < 40 mm.^21^ Massive hemorrhage was defined as the administration of ≥10 units of RBCs within 24 h of admission. Administration of PLT‐t was considered as postresuscitation if administered after 24 h of admission. Platelet count increment was defined as the difference in platelet count before and after PLT‐t and divided by the number of units transfused. CTCOFR was defined as the total number of days before vasopressor therapy, mechanical ventilation, and RRT were all discontinued.^17^, ^18^ A score of 0 was attributed to those patients who did not require any organ support interventions and a score of 15 to those who required any of these interventions for >14 days. Prolonged organ dysfunction was defined as a CTCOFR of >7.^22^ MODS was defined as the presence of two or more SOFA components with a score >2.^19^, ^20^

### 2.6 Data analysis

Continuous variables were reported as median and interquartile range (IQR) and were tested using the Wilcoxon rank sum test and Kruskal–Wallis test with Dunn's post‐test correction for multiple comparisons. Categorical variables were reported as number and percentage and tested using the Fisher's exact test or Chi‐squared test with Bonferroni correction. Multivariable logistic and linear regressions were used to investigate the association between postresuscitation PLT‐t and clinical outcomes of in‐hospital mortality, critical care length of stay, and prolonged organ support, adjusting for age, injury severity, traumatic brain injury, degree of shock, coagulopathy, fluids, PLT‐t, and other blood products administered during resuscitation within the first 24 h of admission. Independently of their statistical significance in the univariable analysis, all variables were included in the multivariable regressions.^23^ Results were reported as odds ratios (OR) or beta coefficients for categorical or continuous variables, respectively, with 95% confidence intervals (CI). Variable inflation factor was used to quantify multicollinearity. We focused our analysis on the subcohort of patients with moderate–severe thrombocytopenia, considering that almost all postresuscitation PLT‐t were administered to these patients. A complete case analysis was performed. A two‐tailed *p*‐value of <.05 was considered significant. Analysis was performed using Prism v10.4.1 (GraphPad Software Inc.) and R v4.1.3 (R Core Team).

## 3 RESULTS

A total of 803 patients were included in the study, of which 109 (14%) patients received at least one PLT‐t in the postresuscitation period. Compared to patients who did not receive a postresuscitation PLT‐t (*n* = 694), those administered a postresuscitation PLT‐t were more severely injured (ISS 34 [22–43] vs. 26 [17–36], *p* < .001), had higher admission base deficit (9 [5–17]mmol/L vs. 4 [1–7]mmol/L, *p* < .001), and were more likely to require a massive transfusion (41% vs. 6%, *p* < .001) and PLT‐t (74% vs. 32% *p* < .001) during resuscitation (Table 1). The postresus PLT‐t group had a higher incidence of moderate (65% vs. 32%, *p* < .001) and severe (28% vs. 3%, *p* < .001) thrombocytopenia during the critical care stay. Rates of MODS (60% vs. 36%, *p* < .001) and prolonged organ support (62% vs. 34%, *p* < .001) were higher in those who received postresus PLT‐t, but there was no statistically significant difference in in‐hospital mortality (22% vs. 16% *p* = .091).

**TABLE 1:** Characteristics of the study cohort stratified by the administration of postresuscitation platelet transfusions.

	Whole cohort (*n* = 803)					Moderate–severe thrombocytopenia (*n* = 341)				
	No PLT transfusion		PLT transfusion		*p*	No PLT transfusion		PLT transfusion		*p*
	(*n* = 694)		(*n* = 109)			(*n* = 239)		(*n* = 102)		
Admission characteristics										
Sex, male	564	(81)	87	(80)	.719	196	(82)	81	(80)	.574
Age, years	36	(25 to 55)	39	(27 to 54)	.466	34	(24 to 53)	39	(27 to 53)	.241
Glasgow coma score	11	(6 to 15)	11	(4 to 14)	.349	12	(6 to 15)	10	(4 to 14)	.097
Base deficit, mEq/L	4	(1 to 7)	9	(5 to 17)	**<.001**	6	(3 to 10)	9	(5 to 17)	**<.001**
SBP, mmHg	118	(94 to 140)	108	(80 to 130)	.**008**	110	(87 to 133)	104	(76 to 130)	.319
INR > 1.2	117	(21)	37	(43)	**<.001**	60	(31)	35	(43)	.062
EXTEM A5, mm	41	(35 to 46)	35	(30 to 41)	**<.001**	36	(31 to 42)	35	(30 to 41)	.346
PLTTEM A5, mm	30	(26 to 34)	27	(24 to 31)	**<.001**	28	(24 to 32)	27	(24 to 31)	.272
Injury characteristics										
Blunt mechanism of injury	536	(77)	86	(79)	.699	183	(77)	81	(79)	.565
Injury severity score	26	(17 to 36)	34	(22 to 43)	**<.001**	27	(19 to 38)	35	(24 to 45)	.**011**
Traumatic brain injury	319	(47)	42	(40)	.208	95	(40)	42	(43)	.639
AIS head/neck ≥3	352	(52)	44	(42)	.068	105	(44)	44	(45)	.921
AIS face ≥3	22	(3)	11	(9)	.**006**	6	(3)	10	(10)	.**008**
AIS thorax ≥3	366	(53)	57	(54)	.812	132	(55)	54	(55)	.983
AIS abdomen ≥3	147	(22)	38	(37)	**<.001**	66	(28)	37	(38)	.064
AIS extremity ≥3	213	(31)	67	(62)	**<.001**	110	(46)	64	(63)	.**004**
AIS external ≥3	18	(3)	1	(1)	.495	7	(3)	1	(1)	.446
Fluids and BP in first 24 h										
Crystalloids, L	3.0	(1.8 to 4.0)	3.9	(2.3 to 5.0)	**<.001**	3.1	(2.2 to 4.4)	4.0	(2.5 to 5.0)	.066
Red blood cells, units	2	(0 to 5)	8	(4 to 15)	**<.001**	4	(2 to 8)	8	(4 to 15)	**<.001**
Massive transfusion	43	(6)	44	(41)	**<.001**	32	(14)	43	(43)	**<.001**
Fresh frozen place, units	0	(0 to 4)	7	(4 to 14)	**<.001**	4	(0 to 8)	7	(4 to 14)	**<.001**
Platelets, pools	0	(0 to 1)	1	(0 to 2)	**<.001**	1	(0 to 1)	2	(1 to 2)	**<.001**
Cryoprecipitate, pools	0	(0 to 2)	2	(0 to 5)	**<.001**	1	(0 to 2)	2	(0 to 5)	**<.001**
Platelet count										
Admission	218	(179–265)	174	(133–211)	**<.001**	204	(158–240)	173	(135–208)	**<.001**
At 24 h	150	(114–190)	90	(73–113)	**<.001**	104	(87–130)	90	(72–108)	**<.001**
Thrombocytopenia in critical care					**<.001**					**<.001**
None	176	(25)	1	(1)		‐	‐	‐	‐	
Mild	279	(40)	6	(6)		‐	‐	‐	‐	
Moderate	219	(32)	71	(65)		219	(92)	71	(70)	
Severe	20	(3)	31	(28)		20	(8)	31	(30)	
Outcomes										
MODS	200	(36)	50	(60)	**<.001**	83	(46)	49	(63)	.**012**
Venous thromboembolism	37	(5)	9	(8)	.222	17	(7)	9	(10)	.586
CTCOFR	3	(1–9)	10	(3–15)	**<.001**	4	(1–12)	10	(3–15)	**<.001**
CTCOFR >7	199	(34)	53	(62)	**<.001**	71	(38)	51	(65)	**<.001**
Critical care length of stay	7	(3 to 16)	15	(8 to 28)	**<.001**	8	(4 to 17)	6	(8 to 28)	**<.001**
In‐hospital mortality	108	(16)	24	(22)	.091	50	(21)	23	(23)	.737

*Note*: Data presented as median (interquartile range) or count (percentage). Thrombocytopenia in critical care was defined as mild (100–149 × 10^9^/L), moderate (50–99 × 10^9^/L) and severe (<50 × 10^9^/L) according to the worst platelet count recorded during the critical care stay. *p* < .05 are hghlighted in bold.

Abbreviations: AIS, abbreviated injury severity score; BP, blood products; CTCOFR, composite time to complete organ failure resolution; INR; international normalized ratio; MODS, multiorgan dysfunction syndrome; PLT, platelet; SBP, systolic blood pressure.

**FIGURE 1:**
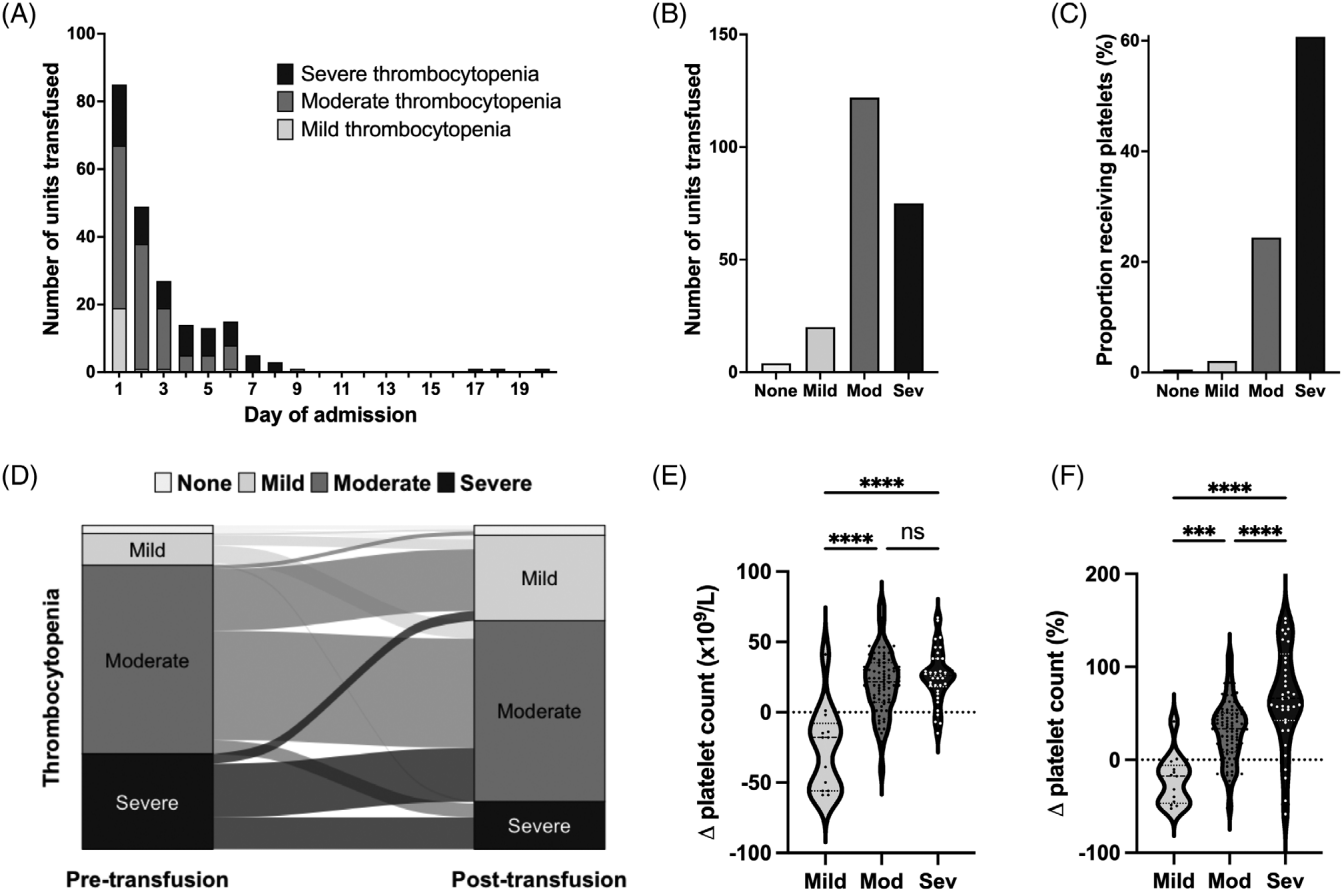
PLT‐t utilization and impact on platelet count trajectories. (A) Number of PLT‐t unit transfused according to the day of admission and severity of thrombocytopenia. (B) Number of PLT‐t unit transfused according to the severity of thrombocytopenia. (C) Proportion of patients receiving PLT‐t according to the severity of thrombocytopenia. (D) Sankey plot depicting thrombocytopenia patterns before PLT‐t (left column) and after PLT‐t (right column). (E, F) Increment in platelet count following PLT‐t according to the severity of thrombocytopenia. None, no thrombocytopenia; Mild (100–149 × 10^9^/L); Moderate or Mod (50–99 × 10^9^/L); and Severe or Sev (<50 × 10^9^/L).

Across the whole study cohort, there were 170 postresuscitation PLT‐t episodes for a total of 221 PLT‐t units, which accounted for 30% (221/725) of all platelet units administered, with the remainder being given during the initial resuscitation phase. The majority of postresuscitation PLT‐t units (160/221, 72%) were given within the first 72 h of admission to critical care and 95% (209/221) were given in the first week (Figure 1A). Overall, the majority of units were given to patients with moderate thrombocytopenia (Figure 1B), although rates of PLT‐t units were highest in those with severe thrombocytopenia (Figure 1C). Preprocedural transfusion accounted for 63% (139/221) of all postresus PLT‐t units. Only 3% (7/221) of all PLT‐t units and 6% (5/82) of nonprocedure related PLT‐t units were given to patients with a platelet count <20 × 10^9^/L, whereas 66% (146/221) of all PLT‐t units and 59% (48/82) of nonprocedure related PLT‐t units were given to patients with a count of ≥50 × 10^9^/L.

Platelet count was available for 163/170 of the PLT‐t episodes. The overall median platelet count increment after PLT‐t was 19 × 10^9^/L (IQR 1–30) per platelet unit transfused. We observed significant variability in the increment according to the degree of pretransfusion thrombocytopenia (Figure 1D), with transfusions given for moderate–severe thrombocytopenia generally resulting in a greater absolute and relative increment (Figure 1E,F). We also found variation in increment by day of administration, with PLT‐t given later after admission generally resulting in a greater positive increment (Figure S1). Almost a quarter (23%, 37/167) of postresuscitation PLT‐t episodes did not improve platelet count. Of PLT‐t episodes that successfully improved the platelet count, 97% (*n* = 122/126) were given for moderate–severe thrombocytopenia as compared to 68% (25/37) of those that did not improve the platelet count (*p* < .001). PLT‐t episodes associated with a positive increment were also more likely to be the first PLT‐t given in the postresuscitation period (PLT‐t with positive increment: 72%, 91/126, vs. PLT‐t with negative increment: 38%, 14/37, *p* = .001).

We next investigated the clinical outcomes associated with postresus PLT‐t. For this analysis, we examined the subgroup with moderate–severe thrombocytopenia (*n* = 341), given that this group accounted for the majority (94%) of patients who received a postresuscitation PLT‐t. Similar to the whole cohort, patients in this subgroup who received a postresus PLT‐t (*n* = 102) were more severely injured (ISS 35 [24–45] vs. 27 [19–38], *p* = .01) and had a higher base deficit on admission (9 [5–17]mmol/L vs. 6 [3–10]mmol/L, *p* < .001) (Table 1). Patients who received a postresuscitation PLT‐t had higher rates of MODS, as well as longer critical care length of stay and need for organ support (Table 1). However, in‐hospital mortality rates were not different (23% vs. 21%, *p* = .737). In multivariable analysis, postresuscitation PLT‐t was associated with a significant reduction in odds of mortality (adj. OR 0.42 [95% CI 0.19–0.96], *p* = .039; Table 2). Among survivors, postresuscitation PLT‐t was associated with a longer critical care length of stay (adj. coefficient 0.35, [95% CI 0.10–0.60], *p* = .007) and prolonged organ support (adj. OR 2.82 [95% CI 1.36–5.84], *p* = .005; Table 2). These findings were replicated when this analytical approach was applied to the entire study cohort (Table S1).

**TABLE 2:** Multivariable logistic regression analysis for in‐hospital mortality, critical care length of stay, and prolonged organ support (CTCOFR > 7) in the moderate to severe thrombocytopenia cohort.

	In‐hospital mortality						Critical care length of stay						Prolonged organ support (CTCOFR > 7)					
	OR	(95% CI)	*p*	Adj. OR	(95% CI)	*p*	Coef.	(95% CI)	*p*	Adj. Coef.	(95% CI)	*p*	OR	(95% CI)	*p*	Adj. OR	(95% CI)	*p*
Age, years	1.03	(1.01 to 1.04)	.**001**	1.04	(1.02 to 1.07)	**<.001**	0.01	(−0.00 to 0.01)	.091	0.01	(−0.00 to 0.01)	.075	1.00	(0.99 to 1.02)	.805	1.00	(0.98 to 1.02)	.970
Injury severity score	1.04	(1.02 to 1.06)	**<.001**	1.01	(0.99 to 1.04)	.321	0.03	(0.02 to 0.03)	**<.001**	0.02	(0.01 to 0.03)	**<.001**	1.06	(1.04 to 1.08)	**<.001**	1.03	(1.01 to 1.06)	.**010**
Traumatic brain injury	3.63	(1.98 to 6.67)	**<.001**	6.00	(2.64 to 13.6)	**<.001**	0.62	(0.38 to 0.85)	**<.001**	0.45	(0.20 to 0.70)	**<.001**	7.12	(3.81 to 13.3)	**<.001**	6.06	(2.89 to 12.7)	**<.001**
Base deficit, mEq/L	1.04	(1.01 to 1.08)	.**013**	1.05	(1.00 to 1.10)	.067	0.01	(−0.01 to 0.02)	.413	−0.00	(−0.02 to 0.01)	.636	1.02	(0.99 to 1.06)	.225	1.03	(0.98 to 1.08)	.282
EXTEM <40 mm	3.16	(1.48 to 6.75)	.**003**	3.08	(1.26 to 7.54)	.**014**	0.09	(−0.15 to 0.34)	.453	−0.09	(−0.31 to 0.13)	.433	1.27	(0.75 to 2.17)	.376	0.84	(0.43 to 1.63)	.597
Total Fluids and BP in 24 h, L	1.10	(1.04 to 1.16)	.**001**	1.16	(1.06 to 1.26)	.**001**	0.04	(0.01 to 0.07)	.**004**	0.03	(0.00 to 0.07)	.**033**	1.04	(0.97 to 1.11)	.246	1.04	(0.95 to 1.13)	.456
PLT‐t 24 h	1.52	(0.82 to 2.79)	.178	1.11	(0.48 to 2.57)	.805	0.14	(−0.10 to 0.38)	.252	0.09	(−0.16 to 0.34)	.470	0.86	(0.51 to 1.45)	.581	0.83	(0.39 to 1.76)	.621
Postresuscitation PLT‐t	1.20	(0.64 to 2.22)	.571	0.42	(0.19 to 0.96)	.**039**	0.63	(0.39 to 0.88)	**<.001**	0.35	(0.10 to 0.60)	.**007**	3.70	(2.03 to 6.75)	**<.001**	2.82	(1.36 to 5.84)	.**005**

*Note*: In‐hospital mortality: *R* ^2^ = 0.24, maximum VIF = 1.74, cases per variable = 7.1. Critical care length of stay: *R* ^2^ = 0.25, maximum VIF = 1.59, cases per variable = 29.3, survivors only. Prolonged organ support: *R* ^2^ = 0.22, maximum VIF = 1.59, cases per variable = 13.9, survivors only. Traumatic brain injury was defined as an AIS Head ≥3. *p* < .05 are hghlighted in bold.

Abbreviations: Adj., adjusted; BP, blood products apart from platelet transfusion; CI, confidence interval; CTCOFR, composite time to complete organ failure resolution; OR, odds ratio; PLT‐t, platelet transfusion.

## 4 DISCUSSION

There are four main findings of this study. First, postresuscitation PLT‐t is common in trauma patients who require admission to critical care, accounting for a significant proportion of the overall transfusion‐related resource burden. Second, there is significant variation in the pretransfusion platelet count, with most patients receiving PLT‐t having only moderate thrombocytopenia. Third, the response to postresuscitation PLT‐t in terms of platelet count is variable, with 1 in 4 transfusions not improving the platelet count. Finally, we found that PLT‐t in the postresuscitation phase appear to confer a survival benefit in trauma patients admitted to critical care but are associated with longer periods of organ dysfunction among survivors.

PLT‐t are a scarce resource that frequently pose challenges to supply chains given the limited shelf life of room temperature stored platelet concentrates.^24^ Overall demand for PLT‐t is increasing in the face of a diminishing donor base, which will exacerbate resource challenges over coming years.^25^ Our results illustrate the extent to which the transfusion‐related resource required to support platelet number and function in major trauma patients extends beyond the resuscitation phase. Much of this resource is consumed within the first 72 h of critical care admission, and a significant proportion of PLT‐t in this phase of care is associated with surgical procedures, often in the context of moderate thrombocytopenia.

Existing guidelines for the use of PLT‐t recommend prophylactic administration to maintain a platelet count >10 × 10^9^/L in the absence of bleeding, or >50 × 10^9^/L prior to major surgery.^25^, ^26^, ^27^ Consistent with previous studies of mixed critical care populations,^11^, ^12^, ^28^ we observed significant variability in pretransfusion platelet counts, with a large proportion having only moderate thrombocytopenia that, according to current guidance, would not usually prompt PLT‐t. Given the lack of randomized trial data on appropriate thresholds, current guidelines acknowledge uncertainty in critical illness and suggest that more liberal thresholds be considered in patients deemed to be at higher risk of bleeding.^25^ Despite this caveat, our results suggest that there may be scope to optimize PLT‐t administration in the trauma population to rationalize resource use and minimize the risks associated with transfusion.

In our study focused on postresuscitation PLT‐t in trauma patients admitted to critical care, we found a median increment in platelet count following PLT‐t of 19 × 10^9^/mL. This relatively modest increment is in line with previous multicenter studies in other patient populations, including mixed inpatient^29^ and critical care populations^28^ as well as postcardiac surgery^30^ patients, which reported median increments ranging from 12 to 23 × 10^9^/mL.^29^, ^30^ A previous study from Stanworth et al. also showed that around a quarter of PLT‐t were ineffectual, with no significant associations with the amount transfused, the presence of ongoing bleeding, or the pretransfusion platelet count. While we found a comparable proportion of PLT‐t failing to improve platelet count, our results also suggest that this failure to improve platelet count occurred more frequently in patients with mild thrombocytopenia or normal platelet count and when administered earlier during the critical care stay. It can be hypothesized that early after injury, multiple factors, such as ongoing bleeding and platelet consumption/destruction, can still contribute to the failure of PLT‐t to improve platelet count.^31^

Perhaps surprisingly, transfusion for profound thrombocytopenia constituted only a small fraction (<5%) of postresuscitation transfusions in this study. We have recently shown that the majority of injured patients admitted to critical care develop some degree of thrombocytopenia during their admission, which consistently occurs between 48 and 72 h after admission and is due to a combination of injury‐related and iatrogenic factors.^13^ When severe, this thrombocytopenia is associated with worse outcomes from organ failure but does not carry an increased risk of mortality, in contrast to other populations. In the present study, we found that PLT‐t given after 24 h were associated with reduced mortality. This suggests a potential therapeutic benefit that may not be entirely dependent on the degree of thrombocytopenia. Interestingly, we also found a longer critical care stay and time to organ failure recovery among survivors who received a postresuscitation PLT‐t. Further studies to define the mechanisms underpinning these observations are important and could potentially guide refinements to the indication for PLT‐t given in nonbleeding critically ill patients.

This study has several limitations. As a retrospective analysis of a prospectively collected dataset, the associations we observed between PLT‐t and clinical outcomes should be interpreted with caution. This was a single‐center study, and thus the generalizability in terms of transfusion practices and patient characteristics may be limited. Finally, we did not specifically collect data on the indication for PLT‐t, and in particular we did not capture bleeding episodes in critical care that would necessitate a therapeutic rather than prophylactic transfusion. However, hemorrhage control procedures were infrequent in our cohort beyond 24 h of injury, and therapeutic PLT‐t in previous studies of mixed critical care patients was uncommon,^11^ so we believe this is unlikely to be a significant contributor to our overall findings.

Overall, this study adds to existing literature highlighting variability in practice and uncertainty regarding indications for PLT‐t outside of bleeding. We have shown that trauma patients consume a substantial PLT‐t resource even after resuscitation, with variable impact on platelet count trajectory. Although the majority of transfusions were given above minimum platelet count thresholds for prophylactic replacement, we found an apparent mortality benefit associated with PLT‐t in this cohort that requires further investigation and validation.

## AUTHOR CONTRIBUTIONS

AR, EC, and PV designed the study. AR, JR, EY, RD, and PV managed, analyzed, and interpreted the data. AR and PV wrote the manuscript. All authors critically revised the intellectual content and approved the final version of the manuscript for publication.

## FUNDING INFORMATION

PV received funding from Wellcome Trust (Early Career Award, 309168/Z/24/Z). The department receives support for rotational thromboelastometry reagents and equipment from TEM International GmbH (Munich, Germany).

## CONFLICT OF INTEREST STATEMENT

The authors have disclosed no conflicts of interest.

## Supporting information


**Data S1:** Supplementary Information.

## Data Availability

The data that support the findings of this study are available from the corresponding author, PV, upon reasonable request.
