# Supplementary material for: Postresuscitation platelet transfusion in major trauma patients
Source: Transfusion. 2025 Sep 19;65(11):2055–64. doi: 10.1111/trf.18414 (PMC12618904; doi:10.1111/trf.18414)
Supplement: Supplementary file 1 — Data S1: Supplementary Information. [file TRF-65-2055-s001.docx]

# Supplemental File


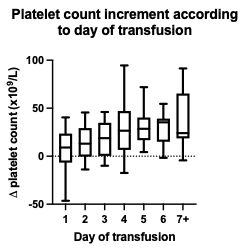


**Supplemental Figure 1.** Platelet count increment according to the day of transfusion.

| **Supplemental Table 1. Multivariable logistic regression analysis for in-hospital mortality, critical care length of stay and prolonged organ support (CTCOFR > 7) in the whole study cohort.** | | | | | | | | | | | | | | | | | | | | | |
| --- | --- | --- | --- | --- | --- | --- | --- | --- | --- | --- | --- | --- | --- | --- | --- | --- | --- | --- | --- | --- | --- |
|  | **In-hospital mortality** | | | | | |  | | **Critical care length of stay** | | | | | |  | **Prolonged organ support** | | | | | |
|  | OR | (95% CI) | p | Adj. OR | (95% CI) | p |  | Coef. | | (95% CI) | p | Adj. Coef. | (95% CI) | p |  | OR | (95% CI) | p | Adj. OR | (95% CI) | p |
| Age, years | 1.02 | (1.01 to 1.03) | **<0.001** | 1.04 | (1.02 to 1.05) | **<0.001** |  | 0.01 | | (0.00 to 0.01) | **0.001** | 0.01 | (0.01 to 0.01) | **<0.001** |  | 1.01 | (1.00 to 1.02) | **0.048** | 1.01 | (1.00 to 1.03) | **0.015** |
| Injury severity score | 1.04 | (1.03 to 1.06) | **<0.001** | 1.01 | (0.99 to 1.03) | 0.264 |  | 0.03 | | (0.02 to 0.04) | **<0.001** | 0.02 | (0.01 to 0.03) | **<0.001** |  | 1.07 | (1.05 to 1.08) | **<0.001** | 1.04 | (1.02 to 1.06) | **<0.001** |
| Traumatic brain injury | 4.89 | (3.05 to 7.83) | **<0.001** | 8.49 | (4.47 to 16.1) | **<0.001** |  | 0.60 | | (0.44 to 0.76) | **<0.001** | 0.43 | (0.27 to 0.60) | **<0.001** |  | 5.92 | (4.09 to 8.57) | **<0.001** | 5.57 | (3.55 to 8.74) | **<0.001** |
| Base deficit, mEq/L | 1.06 | (1.04 to 1.09) | **<0.001** | 1.08 | (1.04 to 1.12) | **<0.001** |  | 0.02 | | (0.00 to 0.03) | **0.007** | 0.00 | (-0.01 to 0.02) | 0.564 |  | 1.04 | (1.01 to 1.07) | **0.008** | 1.04 | (1.00 to 1.08) | **0.028** |
| EXTEM <40 mm | 2.80 | (1.81 to 4.32) | **<0.001** | 2.41 | (1.43 to 4.08) | **0.001** |  | | 0.27 | (0.11 to 0.43) | **0.001** | 0.09 | (-0.06 to 0.24) | 0.234 |  | 1.65 | (1.17 to 2.32) | **0.004** | 1.29 | (0.84 to 1.98) | 0.240 |
| Total Fluids and BP in 24h, L | 1.10 | (1.05 to 1.14) | **<0.001** | 1.13 | (1.05 to 1.20) | **<0.001** |  | | 0.05 | (0.03 to 0.07) | **<0.001** | 0.03 | (0.00 to 0.05) | **0.042** |  | 1.06 | (1.01 to 1.11) | **0.017** | 1.04 | (0.97 to 1.11) | 0.331 |
| PLT-t 24hr | 1.58 | (1.04 to 2.39) | **0.031** | 1.10 | (0.59 to 2.04) | 0.774 |  | | 0.20 | (0.04 to 0.37) | **0.017** | 0.07 | (-0.12 to 0.26) | 0.448 |  | 1.03 | (0.72 to 1.46) | 0.889 | 0.87 | (0.50 to 1.50) | 0.606 |
| Post-resuscitation PLT-t | 1.45 | (0.83 to 2.52) | 0.187 | 0.41 | (0.20 to 0.88) | **0.021** |  | | 0.79 | (0.56 to 1.02) | **<0.001** | 0.45 | (0.21 to 0.68) | **<0.001** |  | 3.90 | (2.30 to 6.61) | **<0.001** | 2.89 | (1.48 to 5.62) | **0.002** |
| In-hospital mortality: R^2^ = 0.24, maximum VIF = 1.90, cases per variable = 13.6. Critical care length of stay: R^2^ = 0.26, maximum VIF = 1.78, cases per variable = 70.9, survivors only. Prolonged organ support: R^2^ = 0.22, maximum VIF = 1.79, cases per variable = 28.0, survivors only. Traumatic brain injury was defined as an AIS Head ≥ 3. CTCOFR, CTCOFR, composite time to complete organ failure resolution; OR, odds ratio; CI, confidence interval; Adj., adjusted; BP, blood products apart from platelet transfusion; PLT-t, platelet transfusion. | | | | | | | | | | | | | | | | | | | | | |
